# Horticultural Crops under Stresses

**DOI:** 10.3390/plants12193400

**Published:** 2023-09-26

**Authors:** Alberto Soares de Melo, Hans Raj Gheyi

**Affiliations:** 1 Center of Biological and Health Sciences, Universidade Estadual da Paraíba, Campus I, Campina Grande 58429-500, PB, Brazil; 2 Center of Tecnologia and Natural Resources, Universidade Federal de Campina Grande, Campina Grande 58429-900, PB, Brazil; hgheyi@gmail.com

Climate change causes alterations in the spatio-temporal temperature and rainfall distribution. These changes have led to soil water restriction and decreased food production. Understanding plant plasticity will result in new genotypes with attributes of interest to farmers and plants adapted to environments under abiotic stresses.

In arid and semi-arid regions, abrupt changes in the soil–plant–atmosphere continuum cause oxidative stress, germination failures, reduced growth, and changes in gas exchange and plant stands, decreasing grains and fruit production. Seedlings grown under water stress conditions associated with high temperatures, a common situation in these regions, have nutrient-uptake restrictions. Another striking phenomenon is increased reactive oxygen species (ROS) production. ROS production changes cell membrane properties and decreases plants’ photosynthetic efficiency. Oxidative stress decreases dry mass production and leaf water status, compromising crop production. Biochemical, physiological, and agronomic responses depend on plant tolerance or sensitivity to abiotic stresses. Thus, studies that explore fitness due to genotypic diversity in hostile environments are needed. Using technologies that enable sustainable agricultural production, even in adverse conditions, will provide farmers with food and economic security.

The literature shows mechanisms for inducing tolerance to abiotic stresses using elicitor application technologies, promoting resilience and stress memory, with biochemical and physiological benefits in plants. *Seed priming* is a technology that presents positive results. *Seed priming* uses several mitigating agents such as silicon, light radiation, gibberellic acid, hydrogen peroxide, and polyethylene glycol 6000. Other technologies had positive results, such as balanced mineral fertilizer for stressful conditions, glycine betaine, salicylic acid, methionine, and bio-input applications aiming to mitigate the harmful effects of salinity, thermal oscillation, and water deficit expected in arid and semi-arid regions.

This Special Edition of *Plants* comprises 12 articles, highlighting the promising results of agrotechnologies in inducing tolerance and diagnosing the nutritional status of plants under abiotic stress. They provide knowledge of plants’ physiological and biochemical processes in challenging environments and elucidate the response mechanisms of elicitors for agriculture. However, many knowledge gaps in knowledge of regarding horticultural cropping systems, especially under adverse conditions, require further investigation due to the environmental dynamics imposed on plants.

In summary, this collection reflects the efforts of multiple researchers in the field of plant sciences, who collaborated with different insights to investigate horticultural plants’ responses under abiotic stress conditions. Therefore, this Special Issue will contribute to instigating new studies that enable the incorporation of scientific and technological knowledge into production processes in the face of climate change.

